# A Practical Diagnostic Approach to Non-Drowning Asphyxia in Animals: Forensic Pathology and Biomarkers

**DOI:** 10.3390/vetsci13030296

**Published:** 2026-03-21

**Authors:** Vittoria Romano, Davide De Biase, Valeria Russo, Evaristo Di Napoli, Orlando Paciello, Giuseppe Piegari

**Affiliations:** 1 Department of Veterinary Medicine and Animal Production, University of Naples Federico II, 80137 Naples, Italy; vittoriaromano119@yahoo.it (V.R.); evaristo.dinapoli@unina.it (E.D.N.); paciello@unina.it (O.P.); giuseppe.piegari@unina.it (G.P.); 2 Department of Pharmacy, University of Salerno, 84084 Salerno, Italy; 3 Istituto Zooprofilattico Sperimentale del Mezzogiorno, 80055 Naples, Italy

**Keywords:** hypoxic injury, carbon monoxide poisoning, post-mortem diagnosis, comparative forensic pathology, sudden death, forensic biomarkers

## Abstract

The term asphyxia refers to a disruption in brain function due to rapid and persistent cerebral hypoxia or anoxia as a consequence of accidental or non-accidental injury. Considering the different mechanisms that may determine asphyxiation, such injuries can be referred to different categories: strangulation (death by hanging, ligature or manual strangulation), suffocation (smothering, choking, confined spaces and vitiated atmosphere), mechanical asphyxia (positional and traumatic asphyxia) and drowning (submersion or immersion in liquid). In both human and veterinary forensic practice, fatal asphyxia is considered among the most diagnostically challenging categories of sudden death, as it often produces only subtle and non-pathognomonic macroscopic signs, which can be easily covered by post-mortem alterations. Therefore, a wide range of information is often needed for the diagnosis of asphyxiation, including medical history, crime scene analysis, testimonies and physical evidence, along with the macroscopic and histological findings. The following review addresses the main lesions, ancillary tests and diagnostic issues associated with non-drowning asphyxia in veterinary forensic pathology.

## Simple Summary

Cases of animal abuse represent a serious yet vastly underreported problem. Among these, the diagnosis of non-drowning asphyxia is particularly challenging in the field of veterinary forensics, often requiring a careful and thorough evaluation of the crime scene, testimonies, circumstances surrounding death and the associated lesions. This review offers a comprehensive analysis of the main pathological findings, diagnostic issues and recent advancements in ancillary tests associated with non-drowning asphyxia in animals.

## 1. Introduction

Asphyxiation can occur in any condition interfering with the uptake or utilization of oxygen and the elimination of carbon dioxide [1]; this may be determined by lack of oxygen in the inspired air (vitiated atmosphere), obstruction of the air passages (suffocation, strangulation), restriction of chest movements (mechanical asphyxia), or histotoxic anoxia [1,2]. Hypoxia and anoxia are generic terms that indicate, respectively, reduction in or depletion of oxygenation, whereas hypoxemia and anoxemia refer to low levels or absence of oxygen in the blood [2,3]. Ischaemia indicates a reduction in or cessation of blood flow to a certain tissue [4], while histotoxic hypoxia refers to impaired utilization of oxygen in a certain tissue [2,3]. These conditions may occur under a variety of accidental circumstances, including endogenous diseases (for example, anaemia or pneumonia), or secondary to non-accidental injury [2], the latter of which is of interest to forensic pathology. Lesions associated with asphyxia have been vastly documented in medical pathology over the years, comprising numerous case reports, case series, reviews, and book chapters; conversely, the currently available literature in veterinary pathology may be considered lacking in comparison. In 2016, McEven extensively reviewed the main morphologic and physiologic responses associated with cases of asphyxia in animals [2], including information from a few case reports available at the time [5,6,7]. Since then, several case reports [8,9,10,11,12,13] and retrospective studies [14,15,16] further expanded the available literature regarding the main histological and gross pathological findings in asphyxiated domestic and wildlife animal species. Furthermore, the past decade showed an increase in studies identifying and attempting to validate ancillary tests associated with death by asphyxia in animals [8,10,17,18,19,20,21]. The importance of veterinary forensics, to recognize and condemn cases of animal cruelty, has been gaining growing recognition in the past few years, although animal mistreatment is still considered an underreported global issue [22,23]. Beyond safeguarding animal welfare, veterinary forensic investigations play a crucial role in the early detection of broader patterns of violence, as animal abuse is increasingly recognized as a potential indicator of interpersonal and domestic violence. Indeed, different studies have shown a significant relationship between animal abuse and social violence, highlighting the need for a One Health, One Welfare approach [22,23,24,25,26]. In light of these considerations, this review provides an updated and comprehensive analysis of the existing literature on pathological findings associated with animal abuse and the main diagnostic issues associated with non-drowning asphyxia in animals, as well as recent advancements in ancillary tests to aid in differential diagnosis in forensic contexts.

## 2. Asphyxia: Classification and General Findings

In medical forensic pathology, as currently reported in standard textbooks and review papers, the classification of asphyxia is highly variable and far from being uniform [2]. An attempt to standardize the categorization and subtype definition was proposed by Sauvageau and Boghossian (Table 1), which showed asphyxia divided into four main categories, suffocation, strangulation, mechanical asphyxia and drowning, each with further sub-categorizations [27].

Regardless of the classification system, asphyxia should always be considered a mechanism rather than a cause of death [28,29]. In cases of asphyxia, the final cause of death can be attributed to a reduction in the oxygenation of the brain tissues (cerebral hypoxia) sufficient to disrupt brain function followed by cardiac arrest [1,14].

Death due to asphyxia, depending on the originating injury or mechanism, may or may not produce identifiable pathological lesions; even when present, these lesions are considered neither sensitive nor specific indicators of asphyxia [2,14]. The classic signs of asphyxia, as reported in medical pathology, are cyanosis, fluidity of blood, visceral congestion, petechiae and engorgement of the right ventricle [29,30,31]. However, these signs are highly non-specific and common post-mortem findings that may result from other pathological mechanisms.

Petechiae are considered the most indicative lesions of fatal neck compression, determined by impaired venous return from the head, and their development is highly dependent on the intensity and duration of the force of the compression applied [2]. As a result, petechiae may be visible in mucous membranes and tissues anterior to the neck compression, most commonly in the skin, conjunctiva, sclera, oral mucosa and larynx. However, petechiae may also occur in cases of strenuous sneezing and coughing, vasculopathies, and coagulative disorders; furthermore, they may not form at all in cases of neck compression with complete obstruction of the arterial vascular supply and venous return, as blood does not enter the tissues [2]. Visceral petechiae may be observed as a consequence of sudden over-distention and rupture of small vessels [32]. The disposition of the body after death, particularly in cases of hanging, may influence the development of congestion and petechiae due to livor mortis [2].

An increase in fluidity of blood can be observed in asphyxia due to the absence of post-mortem clotting [33]; this finding is considered non-specific, since it may be observed in any case of rapid death and is hypothesized to be related to the increased rate of fibrinolysis because of high agonal catecholamine levels [34].

Pulmonary oedema, associated with haemorrhage, atelectasis and interstitial emphysema, are common macroscopic and histologic findings in animals that have died due to asphyxia; however, they may also be observed in several other conditions [2,14].

The detection of these lesions, together with other non-pathognomonic findings, may assist in determining the cause of death, particularly when supported by ancillary tests. However, the diagnosis of death by asphyxia remains a diagnosis of exclusion based on the combined evaluation of pathological findings and the circumstances surrounding death [32].

## 3. Strangulation

**Figure 1:**
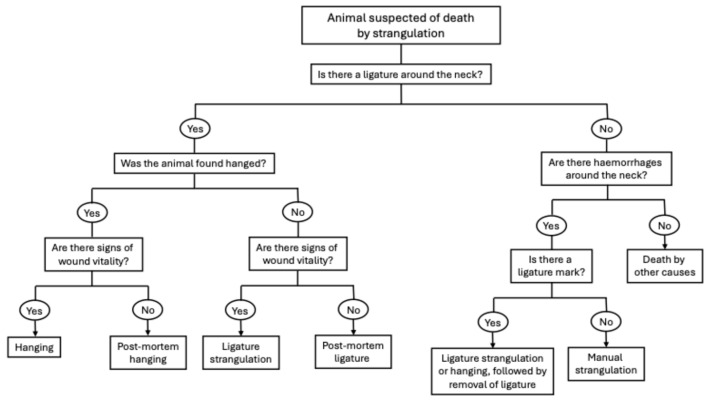
Possible scenarios in case of fatal neck compression.

Strangulation is defined as the compression of blood vessels or air passages of the neck due to external pressure on the neck by a tightening ligature (hanging or ligature strangulation) or manual strangulation (throttling) [27]. The main consequences of strangulation include occlusion of the carotid and vertebral arteries (determining cerebral hypoxia) and airway obstruction by direct pressure on the trachea or due to the displacement of the base of the tongue against the palate [2,32]. The key question, in cases of suspected strangulation, is whether the placement of the ligature around the neck or hanging occurred ante- or post-mortem (Figure 1).

Strangulation in domestic animals may occur due to deliberate abuse or accidentally. For example, accidental strangulation can occur in tethered dogs that attempt to jump over to, or fall off, a platform and become trapped with the leash around the neck. Non-elasticated collars may contribute to accidental strangulation of domestic cats. In equine species, accidental strangulation by neck ropes or head collars may arise from improper animal husbandry practices [1]. An example of deliberate strangulation can be found in the use of a punitive training method known as helicoptering, where the dog is lifted and swung off the ground from the leash [6]. Lesions of strangulation have also been reported in association with cases of animal sexual abuse [35].

The degree and duration of pathophysiological responses to asphyxia may vary widely across species due to differences in vascular anatomy and blood supply to the cerebral tissue. For example, permanent brain damage occurs in humans within 4 min of complete carotid artery occlusion [36], while in dogs, it takes 8 min of total occlusion of the cerebral blood supply [37]. In one experiment, even after 6 min of cerebral ischaemia, dogs successfully recovered to full functionality [37]. The interspecific anatomical differences are important to consider not only in relation to the possible lesions but also pertaining to questions on animal welfare and suffering. Indeed, while compression of the carotid arteries in humans causes rapid unconsciousness [38], ligation of both common carotid arteries in non-anaesthetized dogs, pigs, goats, and calves showed no neurological deficits or behavioural alterations [39]. The maxillary and vertebral arteries contribute significantly to cerebral blood supply in dogs and pigs; similarly, internal carotids in cats are atrophied at birth, and the majority of the blood supply to the cerebral circulation derives from the arterial cerebral circle [32]. Overall, vertebral arteries are more difficult to occlude, and, in addition, the high number of extracranial and intracranial arterial anastomoses makes cerebral ischaemia less common in certain species, such as dogs and cats [2]. Several studies reported prolonged survival in dogs with simultaneous ligation of all common carotid and vertebral arteries [40,41]. Usually, these conditions do not determine cerebral ischaemia in this species unless severe hypotension is also present [41]; therefore, irreversible cerebral damage may only occur when the arterial oxygen levels drop below 20–23 mm Hg and the cerebral blood flow is below 10 mL/100 g/min [42,43]. Overall, these differences result in a prolonged period of struggle in cases of animal strangulation, in part due to the severe air hunger that occurs before the animal loses consciousness. The duration of this period can vary according to the degree of occlusion of the trachea and blood vessels [2]. For example, a dog hung by its chain, with occlusion of both trachea and blood vessels, struggled for approximately one minute before losing consciousness [6]. Conversely, an experiment on tracheal occlusion of unanaesthetised dogs showed periods of struggling ranging from 7 up to 14 min [44].

### 3.1. General Findings of Strangulation

Every subcategory of strangulation is associated with compression of the structures of the neck; therefore, the main gross findings associated with strangulation are localized around the region of the neck, involving the skin, subcutaneous tissue and muscles, as well as the laryngeal and hyoid apparatus. While many of these lesions may be characteristic of strangulation, none of them are pathognomonic [2,14]. Table 2 summarizes the injuries most commonly reported in strangulated animals.

#### 3.1.1. Skin, Subcutis and Muscles

Ligature strangulation and hanging can lead to the formation of linear skin abrasions on the victim’s neck (ligature mark) [34,45], whereas manual strangulation may be associated with the identification of discrete finger marks on the neck [14]. However, these lesions may be absent or less visible in some circumstances. This is due to specific anatomical features, such as the presence of fur, which may protect the underlaying skin from damage caused by friction and compression [14]. Furthermore, external bruising is rarely evident on the skin surface of domestic animals, as canine skin has a relatively reduced blood supply, and natural skin pigmentation may limit the visibility of such lesions in both cats and dogs [32]. Indeed, a retrospective study on cases of asphyxia in domestic animals in the UK showed that cutaneous abrasions on the neck were observed in only 5 out of 29 cases of strangulation; nonetheless, the detection of subcutaneous and muscular injuries in the same region shows a marked increase following complete skinning [14]. Bruises are more common in the cranial half of the neck, particularly in correspondence with the jugular sulcus [14]. Cyanosis may be frequently observed in strangulated animals, particularly at the mucous membranes, teats, perineum and vulva [1,11]. However, it is considered a non-specific finding, since it may be detected in other pathological disorders, such as pulmonary or cardiogenic affections, as well as other categories of asphyxia [1].

#### 3.1.2. Head

Bilateral reddening of the ocular conjunctiva and meninges, associated with lip and tongue bruises, are all changes that may be observed not only in strangulation but also in generalized disorders of blood coagulation [14]; however, the bilateral distribution of said changes may help rule out blunt force trauma to the head, where these alterations are more commonly monolateral [34]. Occasionally, exophthalmos has been reported in cases of strangulation in dogs and cats. This alteration is highly non-specific; it has also been reported as a consequence of smothering [14] and may arise from other pathologies. Haemorrhages and reddening of the sclera are commonly described in both cats and dogs that are victims of strangulation and hanging [14]. In particular, scleral haemorrhages have also been reported in cases of blunt force trauma to the head; therefore, they are not exclusively attributable to strangulation [46]. Furthermore, it has been suggested that they may dissipate during the early post-mortem period and become undetectable at examination [46]. Macroscopic examination of the tongue may indicate the presence of bilateral haemorrhages of the lingual arteries at the base of the tongue or the presence of intramuscular haemorrhage [2,14,32]. Therefore, in cases of suspected strangulation, multiple crosswise slices of the tongue should be performed [32].

#### 3.1.3. Hyoid Apparatus

Hyoid fractures may be difficult to detect on radiographic examination and are sometimes easier to identify at necropsy [32]; neck examination should include careful palpation of the larynx and hyoid apparatus [2]. The ante-mortem nature of the fracture is depicted by the presence of haemorrhage at the fracture site and surrounding tissues [32]. Furthermore, microscopic haemorrhage and fractures may be identified in the laryngeal–hyoid apparatus even when no macroscopic lesions are visible [2]. These fractures are more commonly observed after bone ossification; in carnivores, this occurs two to three months after birth [47]. In dogs, the stylohyoid, thyrohyoid and epihyoid are already ossified at birth; the basihyoid and ceratohyoid ossify after one and two months postpartum, respectively; the tympanohyoid does not ossify [48]. Although hyoid alterations have been occasionally reported in strangulated animals, a recent retrospective study has uncovered a low incidence of these injuries in domestic animals; in particular, this study identified no fractures and only one case of dislocation in a total of 29 evaluated animals (25 dogs and 4 cats) [14]. Conversely, these injuries are frequently reported in human medicine [34]. The lower incidence of hyoid fractures in animals compared to humans may be due to differences in anatomical conformation among species, particularly neck length and diameter [14].

#### 3.1.4. Upper and Lower Airways

Larynx alterations are commonly reported in the forensic literature, mostly consisting of congestion, oedema, and haemorrhages [1,2,7,14,32]. Resuscitative efforts involving intubation may cause similar injuries to the pharynx and larynx; therefore, consideration of the circumstances surrounding death and medical history of the animal is important [32]. The observation of bruising on the distal third of the trachea, over the trachealis muscle, has also been described in cats and dogs (predominantly large-sized) that were either strangled or hanged [14]. The lungs generally appear congested and show irregular areas of collapse (atelectasis); in some cases, they may appear overinflated, with well-defined rib markings [1,2,12,14,15,16,20]. The most common microscopic findings include pulmonary oedema, over-distension of the alveoli, alveolar collapse and intra-alveolar haemorrhage. Free blood within the bronchi and bronchioles may be observed [1,2,12,14,15,16,20]. An important differential feature is the absence of a significant inflammatory cell response in the interstitium and alveoli [1].

### 3.2. Ancillary Tests and Forensic Biomarkers

Few studies have aimed to identify biomarkers and immunohistochemical staining techniques that could support the diagnosis of strangulation. However, most of them have been conducted in human and laboratory animal models [20,24,49,50,51,52,53], as summarized in Table 3.

Wu et al. demonstrated, using mouse models, that ATR-FTIR spectroscopy, combined with machine learning algorithms, can aid forensic pathologists in distinguishing between cases of strangulation and drowning. The results showed distinct biochemical signatures for strangulation and drowning compared to control groups, with significant changes in the peaks associated with the amine band, protein secondary structures and nucleic acids [20]. Balandiz et al. showed that interleukin-1β immunostaining of epidermal cells can be used to discriminate ante-mortem and post-mortem hangings in experimental rat models, showing positive staining in all ante-mortem hanging cases, even 72 h post-mortem [21]. In the human forensic literature, Wang et al. aimed to discriminate between deaths due to different mechanisms of asphyxia (strangulation, smothering and choking) and acute cardiac events through the evaluation of expressed levels of aquaporin 5 (AQP5) in the lungs. The authors identified partial differences in pulmonary molecular pathology among these causes of death, indicating a suppressed expression of AQP-5 in smothering and choking compared to strangulation and sudden cardiac death [50]. Furthermore, Palmiere et al. reported increases in blood concentrations of thyroglobulin, total T3, and free T3 in individuals with fatal mechanical neck compression [51]. Zhang et al. also investigated the expression of two heat shock proteins (HSP27 and HSP70) in human cases of hanging, ligature and manual strangulation, showing significant increases compared to the control cases [52]. The vitality of ligature marks, in human cases of suicidal hangings, has also been evaluated by Caputo et al., showing a significant increase in expression of P-selectin, FVII and MRP8 compared to uninjured skin [53]. This far, the applicability of specific biomarkers to aid the diagnosis of strangulation in veterinary forensics has not been evaluated; even in the human field, these biomarkers are far from being included in routine forensic casework. However, the encouraging results obtained in rat and mice models allow for hypothesizing a possible application in animals as well. Future studies should aim to investigate their validity in different animal species and their specificity and sensitivity in different cases of asphyxia compared to other causes of sudden death.

### 3.3. Diagnostic Limitations

Significant limitations can be encountered in forensic cases of suspected strangulation, mainly as a consequence of its non-specific and highly variable pathological presentation. The complexity of diagnosing asphyxia has been noted in a case report recently published by Huang et al. [12]. This study described a forensic case in which a cat was manually strangulated and subsequently enclosed in an airtight bag; the animal was finally retrieved at the lakeside, with soaked fur. The sequence and chronology of the events were testified by the retrieval of video footage. The main autopsy findings were consistent with manual strangulation, comprising subcutaneous haemorrhages and bruising in the region of the neck. However, the remaining assessed lesions (including marked congestion and overinflation of the lungs) were highly non-specific. Therefore, the authors deemed the final cause of death as inconclusive, emphasizing the impossibility of ruling out death by drowning or suffocation due to vitiated atmosphere [12].

### 3.4. Forensic Approach to Cases of Suspected Strangulation

**Figure 2:**
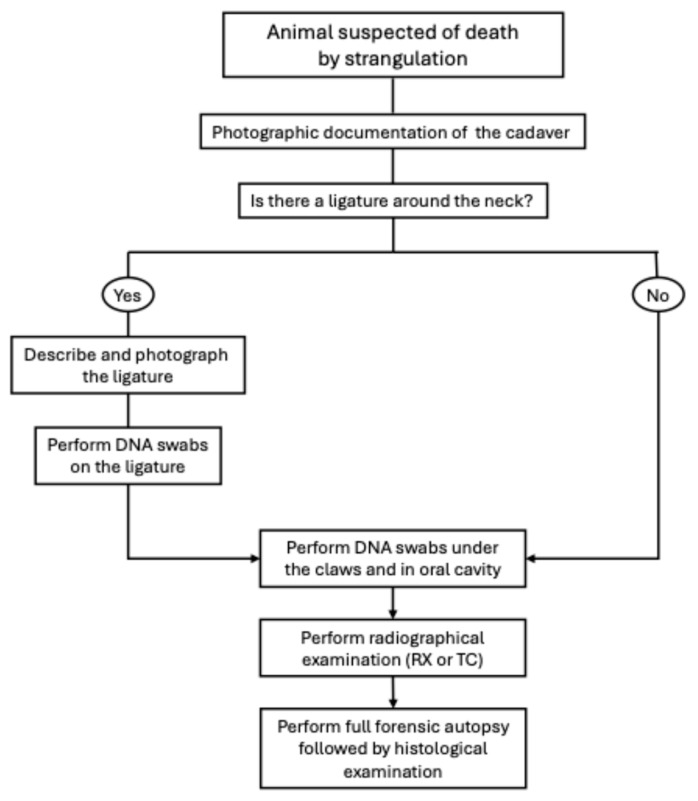
Flowchart of the forensic approach to cases of suspected death by strangulation.

The forensic approach that should be applied in cases of hanging, ligature and manual strangulation is similar (Figure 2). The first step should always be to document photographically the cadaver on retrieval, before any alteration in the crime scene; particular attention should be given to the photographic documentation of the ligature, if present. DNA swabs should be performed on the ligature (if present), claws and oral cavity; indeed, during the initial period of struggling, the animal may have attempted to attack its assailant, thereby possibly retaining important evidence [2]. If the animal was found hanging, care should be taken in the retrieval of the cadaver, cutting the ligature while preserving any existing knots [2]. Before proceeding with the necropsy, radiographic examination (RX or TC) should be performed, particularly to search for possible hyoid fractures or lesions to the cervical vertebrae and to rule out the presence of ballistic material in the cadaver. At this point, the ligature should only be removed from the neck after a thorough photographic documentation and description of the location, type of knot and mode of application. It is preferable to slip the ligature over the neck; when this is not possible, it is recommended to cut the ligature by preserving the original knot, and the cut edges should be secured with string [33]. The ligature should then be placed in a sealed paper evidence bag [2]. Finally, the necropsy should be performed following standardised forensic protocols [54], including full skinning of the cadaver to search for haemorrhages and evidence of fatal neck compression. In addition, particular attention should be given to the examination of the superficial and deep structures of the neck. Removal and fixation of the larynx and trachea, followed by serial sectioning, allow for further in-depth examination [55].

### 3.5. Hanging

Hanging occurs when pressure to the neck is applied by a constricting band that is tightened by the gravitational weight of the body or part of it [27]. The resulting force applied to the structures of the neck may or may not determine obstruction of the airway by compression of the trachea, or through displacement of the tongue when the noose is applied above the larynx [32,34]. When the airway is obstructed, it usually results in a violent struggle (air hunger) [33]. These violent, struggling movements may create hyperextension, hyperflexion and rotational forces that can cause lesions to the cervical vertebrae, such as vertebral displacements, dislocations or even fractures. The compression caused by the ligature may also result in fractures of the transverse processes as well as hyoid fractures [32].

The main findings in hanging victims are reported in Table 4 and may be present either in isolation or in association with the general lesions of strangulation described in Table 2.

In medical pathology, one of the most indicative alterations described in hanging is deemed to be the pooling of blood across the distal extremities and dependent structures of the body. Despite this, it has only occasionally been reported in the veterinary forensic literature [14]; in a retrospective study, conducted on a total of three hanged dogs, pooling of blood was identified in only one case. The main explanation for this low prevalence lies in the longer time required to form a persistent, visible blood pool in the subcutis of the dependent portions of the body. Conversely, the same study reported a 100% prevalence of renal congestion in the assessed dogs [14].

Other findings reported in domestic animals, following strangulation by hanging, include a ligature mark on the skin, associated with subcutaneous haemorrhages around the neck diameter, as well as facial and peritracheal oedema [1,2,14,16]. The ligature mark is considered a highly specific finding of both ligature strangulation and hanging [1,2,14,16,32]; in the latter, the constricting band causes a furrow that generally does not completely encircle the neck (as in ligature strangulation) but rather creates an inverted V-pattern, similar to manual strangulation [32]. This lesion, however, can sometimes be less evident or even absent, depending on the type of ligature used, the duration and the characteristics of animal fur [1,2,32]. Generally, the mark is more prominent when narrow and hard ligatures are used and when the body is suspended for a prolonged period of time due to marked vessel congestion [2,32]. In animals, the fur and looseness of the skin around the neck may protect against skin abrasion [1,2]. Careful examination can identify areas of indentation or patchy hair loss, associated with reddening or mild bruising of the skin [1]. If possible, the fur should be shaved before proceeding with the necropsy exam [2]. The ligature mark may be more visible on reflected skin, in the dermis or subcutaneous tissues [32], and it is important to proceed with a thorough and complete skinning of the animal [2,14,32]. Care should be taken in the identification of signs of vitality to differentiate between ante- and post-mortem hanging; occasionally, animals might be hung after dying from other causes as a consequence of human dysfunctional behaviour. The tissues beneath the noose should be examined for haemorrhage within the skin and underlying tissues, including those around laryngeal fractures, which are indicative of active blood flow at the time the animal was hung [33]. The rest of the body should be further examined for underlying injuries, including claw marks and abrasions, which may result from the animal’s initial struggle. It is also common for hanging victims to have suffered other injuries, before or after hanging, such as blunt or sharp force trauma [32]. Focal intradermal haemorrhages may be observed in the skin of the posterior abdomen and cranially to the ventral vulva [1].

Gross and histological lesions in the lung are non-specific and similar to those found in other cases of strangulation, as summarized in Table 2 [1,2,14,15,16]. Generally, the dependent diaphragmatic lobes appear more severely congested [1].

Similar findings to those described in domestic animals have been reported in a study analysing red fox mortality cases in Madrid, in which one fox was found hanged, and the cause of death was determined to be asphyxia by hanging. The fur around the neck was stained with blood, and the subcutaneous tissue underneath the noose showed a linear pale area, oedema, and focally extensive haemorrhage. Other findings included severe pulmonary emphysema, right ventricle dilation and spleen contraction [16].

### 3.6. Ligature Strangulation (Garrotting)

In ligature strangulation, the structures in the neck are compressed by a constricting band that is tightened by a force other than body weight [27]. The applied pressure causes occlusion of the carotid arteries, reducing blood flow to the cerebral tissues [32].

The ligature may or may not be present on the victim at the time of discovery. If the ligature was removed from the animal, some information regarding the type of ligature that was used may be obtained from the ligature mark. Indeed, the morphology of the ligature mark, when visible, reflects the duration and strength of the force applied, the configuration of the constricting band and its method of application. The mark may, however, be faint or even absent if the ligature was soft or removed immediately after death. A more apparent mark may become evident when narrow and firm ligatures are applied [2,32].

Other general findings are non-specific and may be similar to those found in the case of hanging or manual strangulation. The tissues above the ligature typically appear congested and oedematous; there may be evidence of oedema fluid in the nostrils. Scleral haemorrhage and multiple petechiae on the head, conjunctivae and oral mucosae may be observed [14,32].

### 3.7. Manual Strangulation

Manual strangulation is determined by an external force applied by hands, forearms or other limbs on the structures of the neck [27]. The most common lesions found in the region of the head, when present, are similar to those found in the case of ligature strangulation and hanging; these include congestion, petechiae, cyanosis and oedema fluid in the nostrils [1,2,11,12,14,32]. Indeed, macroscopically evident injuries to the neck structures may or may not be present, depending on the force applied by the assailant and the degree of struggle of the animal. In cases of a large disparity in size between the assailant and victim, the animal may move and struggle very little, showing mild-to-absent haemorrhage [11,32]. Abrasions, contusions or fingernail marks may be identified on skin reflection or deeper tissues [11,32]. Furthermore, during the struggle, the animal may claw at the assailant and possibly retain evidence under its claws [32].

Reddening and bruising are most commonly seen on the dorsal aspect of the larynx, likely because the pressure applied by the thumb of the assailant reaches the back of the larynx due to its intrinsic capacity to move sideways when compressed [14]. The U-shape of the hyoid apparatus makes it susceptible to compression fractures [34], albeit less frequently in animals compared to humans [14].

Rupture of the trachea, associated with findings of subcutaneous emphysema and pneumomediastinum, has also been reported in a dog confirmed dead by manual strangulation [11]. A case report on a manually strangled cat documented luxation at the C6–C7 vertebrae; this finding was associated with haemorrhage in the epidural space and in the surrounding soft tissues [12]. Furthermore, the histologic examination performed in the same study showed tears between the tunica intima and the tunica media of the external jugular vein, as well as a diffuse increase in neuronal satellitosis in the cerebral cortex of the cat [12].

## 4. Mechanical Asphyxia

Mechanical asphyxia occurs when respiratory movements are restricted by external compression on the chest and abdomen (traumatic or crush asphyxia) or by inadequate posture (positional asphyxia) [27].

Traumatic asphyxia is a consequence of compression due to heavy objects and crush injuries of the thorax and abdomen, which determine asphyxia by preventing respiratory movements [2]. Crush asphyxia can also cause death of livestock during transportation in hot weather; in these cases, hyperthermia and smothering may also contribute to the final cause of death [1]. Positional asphyxia may occur in animals hanging in an inverted position, causing the abdominal organs to apply pressure on the respiratory tract and reducing cerebral circulation [2]. When the animal is suspended for a sufficient period of time, death can be the result of acute respiratory or cardiac failure. Additional injuries will be related to the apparatus by which the animal was suspended and its attachment to the body [32].

The forensic approach is similar to the one described for cases of strangulation (Figure 2), and the first step should always be photographic documentation of the cadaver. In this case, diagnostic imaging assumes an even more important role, to evaluate and describe appropriately the traumatic lesions, particularly regarding the skeletal apparatus. The radiographic examination can act as a useful guide during the necropsy exam to find, document and collect accordingly all the traumatic injuries.

Lesions of mechanical asphyxia, if present, are generally considered non-specific. When extreme pressure is applied to the chest, intrathoracic pressure increases significantly, and consequently, venous pressure rises [32]. Therefore, the main findings include congestion and formation of petechiae, which may be difficult to identify due to post-mortem lividity. The formation of petechiae and congestion are directly proportional to the degree of venous obstruction and may be absent if arterial occlusion anterior to the heart is present [2,32,56]. If the animal was bound, the skin may reflect contusions and haemorrhages around the hind limbs [2]. In these cases, the circumstances surrounding death and the pattern of post-mortem hypostasis, in relation to the position in which the animal was retrieved, may aid in the diagnosis of death by positional asphyxia [2]. Other findings may include haemorrhages in chest muscles, rib fractures, internal haemorrhage, and internal organ damage if the animal was a victim of crush asphyxia [2,32].

## 5. Suffocation

Death due to suffocation can be further divided into two main categories: obstructive and non-obstructive suffocation. Obstructive suffocation includes smothering and choking, while non-obstructive suffocation refers to entrapment in confined spaces, vitiated atmosphere and chemical poisoning [27]. There are often few-to-no macroscopic lesions in non-obstructive suffocation, as the animal rapidly becomes unconscious when oxygen is markedly reduced, depleted, or replaced by carbon dioxide [2]. Non-specific findings of lung oedema, emphysema and congestion may be observed [2,14].

Overall, the diagnosis of suffocation is often based on the circumstantial evidence found on the crime scene and possible testimonies. For these reasons, photographic documentation is essential and should always be performed before any manipulation of the cadaver and any physical evidence found at the crime scene. DNA swabs should be carried out on the evidence, as well as under the claws and oral cavity of the animal. Radiographic examination of the cadaver should always be conducted to rule out other possible traumatic lesions before proceeding with the standard forensic necropsy procedures [54].

### 5.1. Smothering

Smothering refers to mechanical obstruction or occlusion of the air passages located above the epiglottis, including the nose, mouth and pharynx [27]. Examples of smothering may include obstruction of the animal’s upper air passages, manual obstruction, the use of objects (such as pillows and plastic bags), or even live burial [1]. Trace evidence of the object or method used may be found in the mouth or air passages of the animal. The main gross findings are representative of the victim’s struggle, including abrasions, contusions or lacerations; there may be evidence of claw marks on the neck if a bag was tied around it and the animal struggled to remove it [14,32]. A study reported a high incidence of bruises over the head, particularly on the labial rim of the mouth, likely due to circumferential compression by the constricting hand of the assailant [14]. There may be petechiae on the pleural surface of the lung and epicardium [14,32]. However, these are all non-specific findings, and the final diagnosis is generally made by exclusion of other causes of death and consideration of the circumstances surrounding the death [32]. The gross and microscopic pulmonary findings are highly non-specific and have a similar presentation to those described in fatal strangulation, including generalised congestion, haemorrhage, oedema, and emphysema [1,14].

### 5.2. Choking

Choking is a consequence of mechanical obstruction of the air passages located below the epiglottis [27]. Death due to choking is often associated with accidental inhalation of small objects during play or eating; however, it may also be related to forcible introduction of foreign material or following live burials [1,32]. Choking may also occur as a result of severe airway swelling secondary to various pathological conditions. Anaphylaxis, blunt force trauma to the neck, and thermal or chemical injuries may lead to swelling of the larynx and surrounding tissues; likewise, inflammatory or neoplastic lesions within or adjacent to the airways may obstruct airflow [32]. Inhalation of blood coming from damaged nasal turbinates, hard palate, skull fractures, or penetrating trauma to the neck can lead to fatal choking due to obstruction of the trachea and lower airways [1].

Diagnosis of choking through necropsy or radiographic examination can be easily made with the detection of foreign material blocking the airway. If the object was removed following resuscitative efforts, the diagnosis can be made based on medical history [32]. Occasionally, acute superficial abrasions in the region of the head may be observed as a consequence of self-inflicted trauma during the agonic period of asphyxia [14].

**Figure 3:**
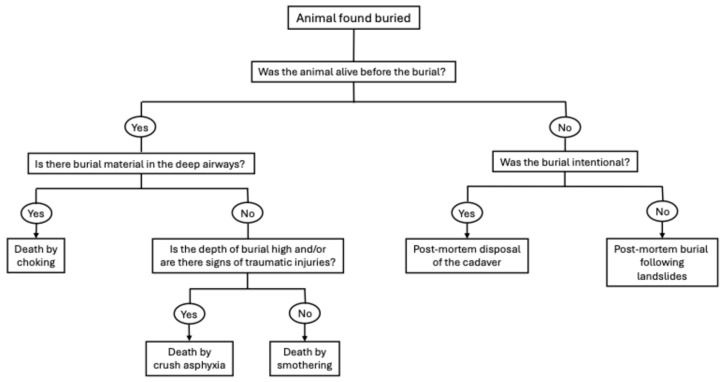
Different scenarios and possible causes of death in case of animals found buried.

The lungs generally show congestion, oedema, intra-alveolar haemorrhage, and alveolar over-distension [14,32]. None of these lesions may be considered pathognomonic; indeed, a retrospective study reported that lung congestion was observed in almost all (4/5) assessed cases of dogs that died from choking, but it did not report this lesion in either of the two assessed cats [14]. The material that caused choking may be found in the bronchi or, if the particles are sufficiently small, in the alveoli [14,32]. In case of live burials, different mechanisms of asphyxia may be involved in determining the final cause of death, as shown in Figure 3.

In relation to the depth of burial and the force impressed on the body, mechanical asphyxia caused by compression of the chest may be the most relevant mechanism involved. Soil may be found in the upper and lower airways as a result of burial material being inhaled or swallowed and may cause choking or smothering of the animal. The presence of small quantities of soil in the outer nares and the mouth may be due to post-mortem soil setting; however, evidence of soil in the nasal cavities, trachea, oesophagus, and stomach indicates respiratory effort and ingestion of soil [32]. Exceptions may occur in the case of catastrophic events such as landslides and earthquakes, where mud may be forced into the deeper air passages of animals that died from other causes [1].

### 5.3. Confined Spaces and Entrapment

Asphyxia in the case of entrapment in confined and enclosed spaces is associated with inadequate quantities of oxygen due to the animal’s gradual consumption of the available oxygen in an airtight container [27,32]. This might occur, for example, when animals are transported in inadequate containers or when they become entrapped in small spaces such as refrigerators or washing machines [1,2,32]. Under these conditions, the cause of death is most likely oxygen deprivation, although hyperthermia and heat stroke may also contribute [1,2]. Necropsy findings are non-specific and include dark fluid blood, epistaxis and generalized organ congestion, associated with oedema of the brain and lungs [14,32]. The identification of pulmonary oedema and congestion, in the absence of underlying natural diseases, provides useful evidence, for example, in investigations of illegal wildlife transportation conducted by law enforcement authorities [1]. No specific ancillary tests are available to confirm death by asphyxia in this context; indeed, carbon dioxide levels rapidly increase after death; therefore, blood analysis for CO_2_ poisoning is not viable for diagnosis [32,33]. Therefore, the diagnosis of asphyxia in enclosed vessels is generally made by exclusion of other causes of death and by considering the circumstances surrounding death [32].

### 5.4. Vitiated Atmosphere and Chemical Asphyxiants

The term vitiated atmosphere refers to a form of non-obstructive suffocation caused by oxygen deficiency, which may be due to displacement by other inert gases (gaseous suffocation) or due to substances that prevent cellular oxygen utilization [27]. The main chemical asphyxiants include carbon dioxide (CO_2_), cyanide (HCN), hydrogen sulphide (H_2_S), polytetrafluoroethylene (PTFE) and carbon monoxide (CO). The pathological lesions and evaluation of certain biomarkers associated with these asphyxiants have been vastly studied in the human medical literature, whereas the current available literature regarding animals mostly comprises research on laboratory animal models. Over the past few years, however, the documentation of carbon monoxide poisoning in companion animals has gained growing attention, as cases of cats and dogs found dead due to house fires are not uncommon. Several studies have aimed to describe not only the main pathological findings but also evaluate the blood concentrations of COHb in cats and dogs to aid in the differential diagnosis in cadavers retrieved from fires [8,9,10,19,20].

#### 5.4.1. Carbon Dioxide Poisoning

Carbon dioxide (CO_2_) is not only an endogenous product of tissue metabolism, which may accumulate in airtight enclosed spaces and cause asphyxia, but it can also act as an exogenous asphyxiant. Elevated levels of CO_2_ may be encountered in industrial settings or in association with combustion, fermentation and putrefaction processes [2]. Fatal hypercarbia in dogs and cats has also been reported due to anaesthetic machine malfunctions [57]. Pathophysiological alterations associated with CO_2_ poisoning are most reported in the literature in consideration of animals intentionally euthanized by this method. Indeed, CO_2_ administration is currently considered the most common euthanasia protocol for laboratory rats and mice [58,59]. However, this method has been recently criticized due to animal welfare issues concerning potential stress and pain during the process. Therefore, several studies have aimed to investigate the biochemical and behavioural responses, as well as the histologic alterations, in CO_2_ administration compared to other euthanasia methods. Overall, these studies showed a lower rate of pathophysiological alterations in alternative euthanasia methods, such as isoflurane, pentobarbital and sevoflurane administrations, compared to CO_2_ [58,59,60,61,62,63]. In particular, the most common histological alterations reported in the literature include lung congestion, haemorrhage, emphysema and atelectasis. Variable degenerative changes in the cardiac muscle may be observed, depending on the time of exposure to CO2 [58]. Furthermore, a comparative study showed mild-to-moderate perivascular and peribronchiolar oedema in mice euthanized by CO_2_ administration; conversely, euthanasia by pentobarbital–phenytoin showed no-to-mild lesions [63].

#### 5.4.2. Cyanide Poisoning

Cyanide (HCN) poisoning can be difficult to prove, as the detection of cyanide in cadavers that have been dead for some time before discovery is often unsuccessful [1]. Indeed, HCN is a highly unstable compound, and if sufficient time passes between death and toxicological analysis, it may no longer be detectable. Generally, cyanide poisoning results in a bright, cherry red unstable colouration of all the tissues and organs. However, this finding is non-pathognomonic, since it may be observed in cases of CO poisoning, and post-mortem changes can easily obscure its detection [1,2,17]. Another relevant indicator, detectable in well-preserved cadavers, is a distinctive bitter almond smell [1,2]. It is important to take note that remnants of cyanide may be inhaled or absorbed percutaneously; therefore, in any suspect case, precautions should be taken before proceeding with the necropsy exam [2].

#### 5.4.3. Hydrogen Sulphide Poisoning

Hydrogen sulphide (H_2_S) is a byproduct of the fermentation of organic matter found in sewer gas [2]. Even small concentrations in the air can be rapidly fatal. Primary findings include cyanosis and dark fluid blood [2,64]. Green discolouration of livor mortis may be observed in cases of hydrogen sulphide poisoning due to the formation of sulphaemoglobin [65]. However, this alteration should not be mistaken with the putrefactive processes that lead to the denaturation of haemoglobin to biliverdin and its reaction with hydrogen cyanide, a putrefactive gas [66]. Neuronal injury caused by H_2_S poisoning was evaluated using animal models (mice). The neurodegenerative lesions involve the thalamus, the inferior colliculus [67], and selective regions of the brainstem, the pons and medulla [68]; the latter are involved in regulating breathing, and, indeed, death by H_2_S poisoning has been attributed to inhibition of the breathing centre [68,69,70].

#### 5.4.4. Polytetrafluoroethylene Poisoning

Polytetrafluoroethylene (PTFE) is a synthetic polymer used in various products, including nonstick cookware, ironing board covers and heat lamp bulbs [71]. Pet birds are susceptible to PTFE toxicosis, mostly as a consequence of overheating frying pans in households [72,73,74]. Cases of PTFE toxicosis have also been reported in poultry [71,75] due to PTFE-coated heat lamp bulbs. The primary gross findings include lung congestion and oedema, moderate dehydration and hepatic lipidosis [71,75]. The main histopathological lesions reported are severe, extensive necrotizing haemorrhagic pneumonitis [76] and multifocal hepatic centrilobular necrosis [71].

#### 5.4.5. Carbon Monoxide Poisoning

Carbon monoxide (CO) is a colourless, odourless and tasteless toxic gas [77]. High levels of carbon monoxide can be produced by faulty heaters, car exhaust or during house fires (fire-related fatalities). CO is rapidly absorbed through the lungs and, having 200–300 times greater affinity for haemoglobin than oxygen, it leads to the rapid formation of carboxyhaemoglobin. Consequently, in fatal CO poisoning, the oxygen levels in the blood are reduced to a point where death occurs from systemic hypoxemia and hypoxia [1,17].

Gross and histological lesions associated with CO poisoning in domestic animals are summarized in Table 5.

The main findings reported in dogs and cats with CO poisoning include bright red discolouration of the mucous membranes, skin and inner surface of the ear pinnae, the aqueous fluid, skeletal muscles and abdominal serosa. As aforementioned, exposure to toxic levels of both CO and HCN may cause cherry red discolouration of the skin in animals, and it may not be considered a pathognomonic alteration [1]. However, carboxyhaemoglobin is a relatively stable compound; therefore, this alteration persists longer and is more commonly detected in CO poisoning than in HCN poisoning [1,2]. In either case, this finding may be obscured by post-mortem alterations; particular care should be taken in the examination of frozen and successfully thawed cadavers, where a generalized pink discolouration of tissues may be observed as a post-mortem artefact [1]. Occasionally, in dogs poisoned by car exhaust fumes, a generalized dark congestion of the mucous membranes can be observed, including injection of the uterine cervix [1]. Other findings include the following: Mild hydrothorax and hydropericardium may also be observed. The lungs generally appear congested and oedematous [8,9,82]. Gross examination of the brain may show cerebral vascular congestion [82], asymmetry in the size of cerebral hemispheres due to uneven dilatation of the lateral ventricles, and a slightly swollen appearance of the cerebrum [78]. However, animals often lack any significant evidence of macroscopic alterations in the brain [9,78].

The main histologic alterations associated with CO toxicity in dogs and cats are generally identified in the caudate nucleus, pallidum, and substantia nigra, with a bilateral distribution [2,78,79]. These lesions may include reactive capillaries and, occasionally, perivascular lymphocyte infiltrates adjacent to neuronal cell bodies showing ischaemic-type degeneration; the same alterations may also be observed, multifocally scattered, throughout the neocortex of all cerebral lobes [78]. Examination of the cerebellum may reveal numerous folia with acute ischaemic-type degeneration of Purkinje neurons, which may also appear reduced in number [78]. Demyelination of deep white matter is described in both humans and cats [80,81]. Despite this, a study on two cats that died from CO poisoning reported the absence of gross and microscopic lesions in the brains of both assessed animals; it was thus hypothesised that a very acute clinical course may leave no time for the development of lesions in the central nervous system [9].

Multifocal intense basophilia of cardiomyocytes has been reported in two cats. This alteration affected entire fibres or, less frequently, only a portion of the fibre, showing a clear line of demarcation from the rest of the fibre [9]. Coagulative necrosis of cardiac myofibres was also described in both humans and cats [79,83].

Finally, microscopic evaluation of lung samples generally shows congestion, interstitial and alveolar oedema [9].

#### 5.4.6. Fire-Related Fatalities and Differential Diagnosis

Fire-related fatalities are defined as any death “that would not have otherwise occurred had there not been a fire” [17]. A variety of lesions may be observed in fire-related deaths, including CO poisoning, often considered the cause of death in FRFs, and thermal injuries, which may have occurred ante- or post-mortem [17]. Indeed, cadavers may be intentionally burned after death as an attempt to destroy evidence of deliberate abuse, such as stab wounds and gunshot injuries [19,84,85]. Therefore, one of the main challenges for pathologists is to determine whether the animal died prior to or during the fire [17]. The main signs of vital exposure to heat and fire fumes are the elevation in COHb% in the blood and a generalized cherry red discolouration of tissues, associated with evidence of soot deposition in the lower respiratory tract and the stomach [19,86].

The blood carboxyhaemoglobin (COHb%) concentration is an important biological marker corroborating ante-mortem COHb inhalation during fires. Physiological and lethal ranges have been reported in the literature in both dogs and cats, as summarized in Table 6.

Physiologically, the normal range of COHb% in dogs is 0.1–6.4% [18,87], and in cats, it is 0.1–4.4% [87]. Mild increases in COHb% are reported due to exposure to environmental pollution and cigarette smoke [17,18]; furthermore, a retrospective study reported that dogs with respiratory diseases showed a slight increase in COHb% compared to those without respiratory disease, while no significant difference was seen between cats with and without respiratory diseases [87]. COHb values measured in dogs that survived a kennel fire ranged from 8.8% to 37% [18]; another study reported levels of COHb% in peripheral blood ranging between 24–76% and 23.9–62.5%, respectively, in adult dogs and puppies that died in fire-related conditions [17]. Interestingly, the same study reported higher COHb concentrations in the lungs of puppies than in peripheral blood; conversely, no significant difference between the two was detected in adult dogs [17]. The correlation between COHb% in pulmonary and peripheral blood is a direct consequence of the duration of the victim’s smoke inhalation. Indeed, puppies are highly sensitive to environmental alterations, being poor regulators of their body temperature, and they have lower respiratory rates compared to adult dogs [88]; slight exposure to high temperatures and smoke can lead to rapid death of young animals, resulting in limited diffusion of COHb in the peripheral bloodstream [17]. Therefore, measuring pulmonary COHb% in puppies is suggested to aid differential diagnosis.

The utility of CO-oximetry has also been evaluated in cats that died due to CO poisoning in a secondary arson homicide. The results of the study showed the following values in four cats: 73.3%, 74.4%, 67.9%, and 66.9% [8]. In another case report, COHb saturation in two cats deemed to have died due to CO poisoning was 57% and 41% [9]. CO poisoning in cats is underreported and scarcely studied in the literature compared to dogs. The few data regarding both the normal and pathological ranges of COHb% arise from a small sample size; therefore, further studies are required to establish proper diagnostic thresholds in this species.

Nonetheless, studies in both humans and animals have demonstrated an increase in COHb% after post-mortem exposure to CO, albeit smaller than ante-mortem levels. Indeed, dogs exposed post-mortem to heat and smoke for 30 min showed an increase in blood COHb% ranging from 3.1% to 18.7% (mean value). Similarly, the concentration of COHb in the lungs ranged between 31.8–75% in adult dogs that died in FRFs, 47–64.1% in puppies that died in FRFs and only 4–13% in post-mortem exposure [17].

The post-mortem passage of CO in cadavers was also evaluated in another study using stillborn piglets with intact or breached skin. Blood samples from the non-exposed piglets indicated basal carboxyhaemoglobin levels around 1%; stillborn piglets without cavity breaches showed no appreciable rise in the COHb levels in blood samples after exposure to 10% CO for two hours. Conversely, stillborn piglets with small cavity breaches showed a minimal rise in COHb levels; larger cavity breaches in piglets exposed to 10% CO for two hours showed an increase in COHb levels to 12% ± 2.6%. This was not observed, however, in heart blood samples [10].

Gross and histological findings may also aid in distinguishing whether the animal died before or during the fire. Indeed, while some lesions may be identified both as a consequence of ante- and post-mortem heat exposure, certain lesions are present solely in the case of ante-mortem exposure to heat, as shown in Table 7.

The main microscopic findings associated with ante-mortem thermal injuries in the skin are elongation of epithelial cells, subepidermal blisters, dermal haemorrhages and necrosis, and neutrophilic infiltration (second- to third-degree burns). Other skin lesions, including homogenization of connective tissue, loss of cellular detail, dermal vacuolization, and detachment of the epidermis, may be visible in both ante- and post-mortem exposure to heat [17]. The mucosa of the pharynx, larynx and upper oesophagus may show oedema and vesicular detachment, which represent signs of vital exposure to heat reported in dogs, along with an increase in the secretion of mucus and pseudo-goblet cell formation [19]. Histological examination of the lungs may show pulmonary oedema and vascular ectasia; however, these are highly non-specific findings that may also be found in post-mortem exposure to heat [17]. Findings specific to ante-mortem exposure include elongation of bronchial epithelial cells and varying amounts of black granular material in the trachea, bronchi, or alveolar walls. This may be associated with evidence of a high number of macrophages in the lungs and pulmonary lymph nodes containing black particles in the cytoplasm [8,9,17,19].

These alterations may vary with the animals’ age. A study reported that puppies subjected to FRFs showed little-to-no soot deposits in the airways at necropsy; only mild-to-moderate amounts of soot were observed histologically [17]. As aforementioned, the physio-morphological characteristics of puppies [88] make them highly susceptible to environmental changes, thereby reducing survival time and limiting soot deposition [17].

## 6. Bodies Recovered from Water, Drowning and Differential Diagnosis

**Figure 4:**
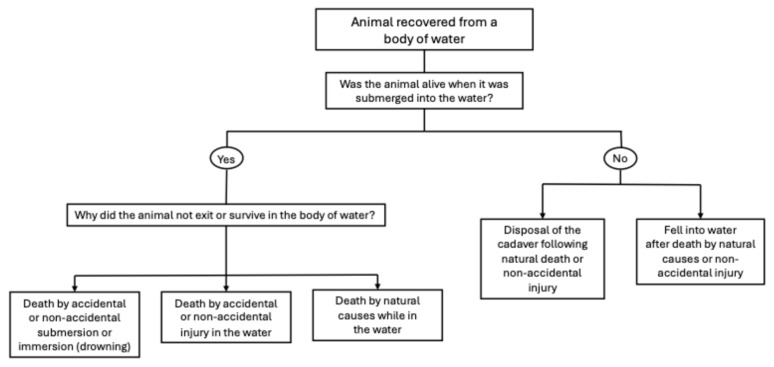
Different scenarios and possible causes of death in cases of animals recovered from bodies of water [90].

The differential diagnosis in cases of bodies recovered from water is complex and challenging in both the human and veterinary forensic literature. Similarly to non-drowning asphyxia, the diagnosis of drowning is still one of exclusion, requiring information from the clinical history of the victim, reliable witness accounts and crime scene or recovery scene analysis. Indeed, tides, currents, people or animals may move the cadaver from the original crime scene, which may even remain unknown [90]. Determining whether the animal was alive when it entered the water is the first key element (Figure 4). Animals may have fallen or have been disposed of in bodies of water following natural or non-accidental death. If the animal was alive when it entered the water, death may have occurred due to drowning or other unrelated causes, such as natural death or accidental and non-accidental injury [90]. Drowning was defined by the World Health Organization in 2002, as “the process of experiencing respiratory impairment from submersion/immersion in liquid”, where the “outcomes are death, morbidity and no morbidity”; in this case, the submersion or immersion may be accidental or non-accidental [91].

Drowning asphyxia is associated with non-specific lesions, reported in Table 8, which are considered common findings associated with several pathological conditions, including non-drowning asphyxia. Indeed, a study showed similar findings between animals that died from drowning and control animals that were experimentally immersed in water. Therefore, necropsy and histological techniques reported a low contribution in determining the final cause of death in animals recovered from bodies of water [92].

To overcome these limits, several ancillary tests have been evaluated to aid in the diagnosis of drowning, particularly in human forensic pathology. These include real-time PCR assays, microbiological and electrolyte assays, and, among all, the one that has received most attention over the years is the diatom test. Diatoms are photosynthetic and autotrophic unicellular organisms which can be found in fresh and saltwater, particularly in stagnant bodies of water. The diatom test relies on the principle that diatoms can be detected in tissues of drowned victims that aspirated diatom-rich water before death. Generally, the size of most diatoms, ranging between 20 and 200 µm, allows them to easily penetrate through the alveolo-capillary barrier, enter the bloodstream and reach highly vascularized organs, such as liver, kidneys, brain and bone marrow. The presence of diatoms in these extra-pulmonary sites is strongly indicative of vital submersion as opposed to post-mortem immersion [90,92,93]. In particular, bone marrow is considered the most reliable substrate for diatom analysis, as it is less exposed to external contamination while the body is in the water [94]. In the last few years, the diatom test has been gaining growing attention in the field of veterinary forensics, and few studies have evaluated their applicability in cats and dogs, as well as cetaceans and sea turtles, showing encouraging results [92,93,95]. Nonetheless, the diatom test’s sensibility and specificity, in both human and veterinary fields, remains controversial. The main limitations rely on potential post-mortem contamination of organs, the possibility of false-positive results due to ante-mortem penetration of diatoms, and even sporadic false-negative results [92]. Indeed, the number and diatom species vary according to annual and seasonal rhythms as well as the habitat and are more common in stagnant waters than in the sea [94]. Despite this variability, the extreme resistance that characterizes diatoms, due to the presence of a siliceous cell wall, makes them unique substrates that are extremely useful in the forensic investigation of cadavers retrieved from water. Furthermore, their specific distribution in the aquatic environment may also provide information on whether the recovery scene corresponds with the location of drowning [94]. Therefore, for a correct analysis, the water in which the cadaver was retrieved should be collected, with samples taken from the surface and from depth in sterile containers and stored at 4 °C [94].

## 7. Post-Mortem Interval Effects on Diagnostic Accuracy

The post-mortem interval (PMI) refers to the range of time between death and examination of the cadaver; in forensic pathology, it is considered among the most important research topics [96]. In veterinary forensic practice, animal cadavers are often discovered after a significant post-mortem interval. After an animal dies, the body undergoes a variety of changes, including livor mortis, rigor mortis, algor mortis and, finally, the different stages of decomposition [65,96]. These processes are affected by different intrinsic and environmental factors, inducing changes in tissues and organs that may obscure pathological findings useful to determine the final cause of death. This is particularly important in cases of asphyxia, where the pathological presentation is highly variable and non-specific. Livor mortis refers to the pooling of blood in the dependent sites of the cadaver due to gravitational forces after the heart stops beating. In animals, lividity is typically most evident in light-coloured skin, mucous membranes and the sclera and may be also observed in internal organs. Livor mortis can obscure the presence and evaluation of several lesions associated with the different categories of asphyxia [2,65]. Furthermore, this post-mortem alteration should be differentiated from congestion or injury. Indeed, lividity should not be mistaken with areas of ante-mortem bruising; the area should be incised and carefully evaluated through macroscopical and histological examination, which can aid in the differentiation. Bruises generally show diffuse oedema and haemorrhage in the surrounding soft tissues, while post-mortem lividity is characterized by blood confined in the blood vessels, at least in the early stages; indeed, the final stages of livor mortis are associated with haemolysis and breakdown of the blood vessel walls, occurring approximately 8 to 12 h post-mortem [65,66,97]. Other important discriminatory elements are the presence of an inflammatory infiltrate or fibrin, which are generally absent in cases of post-mortem lividity, although few studies have demonstrated the sedimentation of neutrophils in large areas of extravasated blood [65,97]. Furthermore, intense areas of lividity may induce the formation of post-mortem “petechiae”. This may be observed in cases of hanging, where gravitational forces cause the pooling of blood to rupture small vessels and cause post-mortem petechiae or larger purpura. This phenomenon is thoroughly reported and described in human forensic pathology, referred to as Tardieu spots, and may occur in as little as two to four hours or up to twenty-four hours [2,65]. However, this lesion is not properly documented in animals; therefore, the term Tardieu spots should be avoided, and in veterinary forensic practice, it is preferrable to describe the distribution and location of the petechiae [2]. Indeed, based on their location, the post-mortem petechiae may be differentiated from the ante-mortem ones; the latter generally extend beyond the areas of lividity and may be located in non-dependent areas of the body [65]. The colour of lividity is generally reddish-to-violet due to oxygen dissociation that continues after death [65], but it can vary due to oxygen content, environmental conditions or certain poisonings. Therefore, the colour of livor mortis is also an important element to consider in forensic practice to aid in the differential diagnosis in cases of suspected asphyxia, particularly in cases of chemical poisoning [65], as shown in Table 9.

Decomposition is another factor that can highly influence diagnostic accuracy in forensic practice; this process involves putrefaction and autolysis. Decomposition may start as soon as six to thirty-six hours, though its onset and rate are highly variable depending on the environment and body condition prior to death [65,66]. Autolysis is a chemical intrinsic process caused by the intracellular enzymes and involves the breakdown of tissues and organs [65,96]. This process is accelerated by heat and in organs rich in enzymes, such as liver and pancreas. Putrefaction involves bacteria, generally spreading from the gastrointestinal tract after death. Indeed, the rate of putrefaction of the abdominal organs is generally higher compared to the rest due to the high bacterial content [98]. For the same reasons, putrefaction is accelerated in the case of sepsis prior to death and may continue even with refrigeration of the cadaver [65]. Environmental conditions also infer with the development of putrefactive processes; the optimal temperatures are between 21° and 38 °C. Extremely high temperatures can delay or slow it down due to heat fixation of tissues and the inactivation of autolytic enzymes [65]. The rate also slows below 14 °C and may stop in extreme cold [65]. Furthermore, putrefaction may be accelerated in animals showing a high body condition score, a heavy fur coat, or that are wrapped, creating heat retention. In fire-related fatalities, decomposition progresses at a faster rate in charred areas compared to areas with light charring [65]. The activity of scavenger animals and insects also plays an important role in the decomposition of cadavers, particularly in certain environmental conditions. This aspect was evaluated in a study on decomposition, conducted by placing five small pig cadavers in different environments (surface deposit with no cover, surface deposit covered with tree branches, enclosed in a carpet, buried in a shallow grave and suspended by a rope) for seventy-five days, with average temperatures ranging between 16 °C and 32 °C. In all cadavers, except the hanging pig, the soft tissues appeared almost completely consumed, consisting of skeletal remains by day twelve. In contrast, the hanging pig was protected from insect activity and scavenging; this, in addition to exposure to sun and wind, caused rapid desiccation and partial mummification of the cadaver [99]. Overall, careful consideration of the post-mortem interval, the position of the cadaver, the environmental conditions and other circumstantial evidence surrounding death are essential to allow for a better understanding and interpretation of both livor mortis and decomposition in relation to the final cause of death.

## 8. Conclusions

This review provides a comprehensive analysis of the existing literature regarding the main pathological findings and post-mortem biomarkers commonly evaluated in asphyxiated animals. In the past, forensic lesions in veterinary textbooks were directly transposed from the medical literature. However, over the past decades, various studies have reported differences in both frequency and pathological presentation of certain findings in different animals compared to human pathology. These studies improved diagnostic accuracy in veterinary forensics. Despite this, a complete characterization of injuries across the different species is far from being fully elucidated, and non-drowning asphyxia remains a challenging diagnosis in veterinary forensic pathology, largely due to its non-specific and highly variable pathological presentation. Indeed, the peculiar anatomical differences found across the species are responsible for different physio-pathological responses to asphyxia in animals and do not allow for a direct translation of the lesions observed in domestic animals across all the species. Therefore, additional studies on wildlife and exotic and farm animals will be needed to further explore the pathological presentation of the different categories of asphyxia in veterinary forensic pathology. Future studies should also give particular attention to the research and implementation of ancillary tests and forensic biomarkers, which are currently little described in veterinary medicine and only evaluated in a limited number of species. In particular, the promising results obtained in mice and rat models, as well as human samples, suggest the possibility of implementing tissue vitality biomarkers in other species to aid in the diagnosis of strangulation. Furthermore, the currently available literature on chemical asphyxia in animals mostly comprises studies on carbon monoxide poisoning, particularly in cats, dogs and, more rarely, pigs. The study and documentation of the pathogenesis, lethal concentrations and associated macroscopic and histologic lesions of other chemical asphyxiants are noticeably lacking in the veterinary field, as most studies have been conducted on human cases. Enhanced awareness, implementation of standardized forensic protocols, and broader application of ancillary diagnostic testing will help pathologists to better recognize and characterize cases of death by asphyxia in domestic and wildlife animals.

## Figures and Tables

**Table 1:** Classification of asphyxia adapted from Sauvageau and Boghossian [27].

Classification	Subcategory	Definition
Strangulation	Hanging	Pressure on the neck applied by a constricting band tightened by the gravitational weight of the body
	Ligature strangulation	Pressure on the neck applied by a constricting band tightened by a force other than body weight
	Manual strangulation	Pressure on the neck applied by hands, forearms or other limbs
Mechanical asphyxia	Positional asphyxia	The position of the individual compromises the ability to breathe
	Traumatic asphyxia	External chest compression by a heavy object
Suffocation	Smothering	Obstruction of the air passages above the epiglottis, including nose, mouth and pharynx
	Choking	Obstruction of the air passages below the epiglottis
	Confined spaces	Environment of inadequate atmosphere by reduction in oxygen
	Vitiated atmosphere	Environment of inadequate atmosphere by displacement of oxygen by other gases, or gases causing chemical interference with oxygen uptake and utilization
Drowning		Asphyxia by immersion in a liquid

**Table 2:** Lesions of strangulation reported in animals.

Tissue or Organ	Lesion
Skin and subcutaneous tissues [1,2,11,12,13,14,15,16,32]	Ligature mark Abrasion, contusion Haemorrhage Subcutaneous emphysema
Muscles [1,2,11,14,15,16,32]	Haemorrhage Contusion Congestion
Eyes [1,2,7,14]	Conjunctival reddening and petechiae Scleral reddening and petechiae Exophthalmos
Meninges [11]	Congestion Oedema
Tongue [1,2,7,14]	Oedema Haemorrhage
Laryngeal–hyoid apparatus [1,2,7,14,32]	Congestion Oedema Haemorrhage Fracture Dislocation
Trachea [1,7,11,14,16]	Rupture Haemorrhage Peritracheal oedema Bruising of the trachealis muscle
Lungs [1,2,12,13,14,15,16,20]	Congestion Atelectasis Emphysema Haemorrhage

**Table 3:** Forensic biomarkers investigated in cases of strangulation asphyxia.

Author and Year	Biomarker Analysis	Species	Number of Samples	Types of Asphyxia Investigated
Wu et al. 2025 [20]	ATR-FTIR spectroscopy	Mice	144	Strangulation vs. drowning
Balandiz et al. 2015 [21]	Immunohistochemical IL-1β antibody staining	Rats	20	Ante-mortem vs. post-mortem hanging
Wang et al. 2012 [50]	Immunohistochemical AQP-5 expression	Human	64	Strangulation, smothering and choking vs. control groups
Palmiere et al. 2018 [51]	Blood concentrations of thyroglobulin, total T3 and free T3	Human	12	Strangulation vs. control groups
Zhang et al. 2023 [52]	Immunohistochemical HSP27 and HSP70 expression	Human	45	Strangulation vs. control groups
Caputo et al. 2023 [53]	Immunohistochemical fibronectin, P-selectin, FVIII, HSP-70 and MRP-8 expression	Human	45	Hanging vs. control groups

**Table 4:** Lesions of hanging reported in animals.

Tissue or Organ	Lesion
Skin and subcutaneous tissues [1,2,14,15,16,32]	Ligature mark Abrasion Pooling of blood across distal extremities Haemorrhage Oedema
Cervical vertebrae [32]	Displacement Dislocation Fracture
Lungs [1,14,15,16]	Marked congestion of dependent lobes Oedema Emphysema Haemorrhage
Kidneys [14]	Congestion

**Table 5:** Lesions of CO poisoning reported in animals.

Tissue or Organ	Lesion
Skin and subcutaneous tissues [8,9]	Cherry red discolouration
Central nervous system [2,78,79,80,81,82]	Congestion Oedema Perivascular lymphocyte infiltrates Neuronal ischaemic degeneration Demyelination of deep white matter
Lungs [8,9,82]	Congestion Oedema
Heart [9,79,83]	Hydropericardium Coagulative necrosis of myofibres Basophilia of cardiomyocytes ^1^

^1^ Observed in cats.

**Table 6:** COHb% saturation in animals.

Subjects	COHb%
Normal range in dogs [18,87]	0.1–6.4%
Dogs that survived a fire [18]	8.8–37%
Adult dogs that died in FRFs [17]	24–76%
Puppies that died in FRFs [17]	23.9–62.5%
Normal range in cats [87]	0.1–4.4%
Cats that died in FRFs [8]	66.9–74.4%
Cats that died from CO poisoning [9]	41–57%

**Table 7:** Gross and histological lesions associated with ante- and post-mortem heat exposure in animals.

Tissue or Organ	Ante-Mortem Heat Exposure	Post-Mortem Heat Exposure
Skin and subcutaneous tissue [8,10,17,19]	Cherry red discolouration Skin charring and splitting Thermal injuries Connective tissue homogenization Loss of cellular detail Dermal vacuolization Detachment of the epidermis Elongation of epithelial cells Subepidermal blisters Dermal haemorrhage and necrosis with inflammatory cells	Cherry red discolouration ^1^ Skin charring and splitting Connective tissue homogenization Loss of cellular detail Dermal vacuolization Detachment of the epidermis
Upper and lower respiratory tract [8,17,19]	Soot deposition Oedema and vesicular detachment (pharynx and larynx) Pseudo-goblet cell formation	Soot deposition (upper tract)
Oesophagus and stomach [8,19]	Soot deposition Oedema and vesicular detachment (oesophagus)	Soot deposition (oesophagus) ^1^
Lungs [8,9,17,19]	Congestion Oedema Elongation of bronchial epithelial cells Black granular material (trachea, bronchi, alveoli) Macrophages containing black material	Congestion Oedema

^1^ Occasionally. The main gross findings associated with ante-mortem exposure to heat and CO include the characteristic cherry red discolouration of the mucus membranes, skin charring, skin splitting and thermal injuries, soot deposition in the upper and lower respiratory tract and, occasionally, bone fractures [8,17,19]. Soot may also be identified in the oesophagus and stomach [8,19]. The lungs generally appear hyperaemic, with moderate-to-severe oedema [8,17,19]. Post-mortem exposure to heat may mimic ante-mortem findings, including skin charring and splitting, as well as soot deposits. However, the distribution of soot deposits is limited to the upper respiratory tract, including the nasal cavity, mouth and larynx [17]. Cherry red discolouration of the skin may occasionally be observed following post-mortem exposure to carbon monoxide. Indeed, it has been shown that oxygen can diffuse through the skin to re-oxygenate blood after death [89], and the passive diffusion of carbon monoxide resulting from post-mortem exposure may determine the local formation of carboxyhaemoglobin [10,17]. Stillborn piglets exposed to 1% carbon monoxide for 30 min showed a visible change in the colour of their hypostasis to cherry red [10]. However, re-oxygenation of the skin can occur only for a limited time after death [89]; this alteration is rarely a consequence of post-mortem exposure [10].

**Table 8:** Lesions associated with drowning.

Tissue or Organ	Lesion
Skin and fur [90,92]	Wet hair coat Contusions
Upper airways [90]	Froth in nostrils, oral cavity or larynx Evidence of foreign material in larynx Mucus in larynx
Lungs [90,92]	Congestion Oedema Haemorrhages Emphysema Evidence of foreign material in alveoli
Heart [90,92]	Right-ventricular distention
Stomach [90,92]	Evidence of foreign material

**Table 9:** Livor mortis discolouration.

Aetiology	Colour of Lividity	Mechanism
Normal	Reddish-purple	Venous blood
Carbon monoxide poisoning	Cherry red/pink	Formation of carboxyhaemoglobin
Cyanide poisoning	Cherry red/pink	Excess of oxygenated blood due to the inhibition of cytochrome oxidase
Fluoroacetate poisoning	Cherry red/pink	Inhibition of oxidative cellular mechanism
Hypothermia, refrigeration, immersion in water	Cherry red/pink	Oxygen retention in cutaneous blood due to cold air or water
Acetaminophen, sodium chlorate, nitrite, naphthalene, phenols, phenazopyridine, skunk spray	Brown	Formation of methaemoglobin
Hydrogen sulphide poisoning	Green	Formation of sulphaemoglobin

## Data Availability

No new data were created or analyzed in this study.
